# Thyroid Hemiagenesis Associated with Hashimoto's Thyroiditis

**DOI:** 10.1155/2013/414506

**Published:** 2013-10-02

**Authors:** D. Nsame, A. Chadli, L. Hallab, S. El Aziz, H. El Ghomari, A. Farouqi

**Affiliations:** ^1^Service d'Endocrinologie, Diabétologie et Maladies Métaboliques, Centre Hospitalier Universitaire Ibn Rochd 1, Quartier des Hôpitaux, Casablanca 20100, Morocco; ^2^Résidante 3ème Endocrinologie, Maladie Métabolique et Nutrition, Centre Hospitalier Universitaire Ibn Rochd 1, Quartier des Hôpitaux, Casablanca 20100, Morocco

## Abstract

Thyroid hemiagenesis is a rare congenital anomaly resulting from failure of one thyroid lobe development. We report a 23-year-old female presented with Hashimoto's thyroiditis in left lobe, associated with hemiagenesis of right lobe and isthmus which was previously diagnosed as Graves' hyperthyroidism, but developed further into Hashimoto's thyroiditis after being treated with antithyroid drugs. The symptoms of hyperthyroidism in the current case led to the diagnostic confirmation by scintiscanning of an absent lobe. The antithyroid pharmacotherapy by thiamazole was used. However, due to symptoms of hypothyroidism, it was discontinued two months later, so thyroid hormone substitution was reintroduced. Antithyroid antibody studies and ultrasonography documented the presence of Hashimoto's thyroiditis.

## 1. Introduction 

Thyroid hemiagenesis is a rare congenital anomaly resulting from failure of one thyroid lobe development. The detection is often made by either clinical symptoms of thyroid dysfunction, by imaginological studies, or by anatomical abnormalities. A variety of pathological conditions occur in the remaining thyroid tissue in association with this rare anomaly such as adenoma, carcinoma, subacute thyroiditis, colloid nodule, Graves' disease, simple goiter, and Hashimoto's thyroiditis. Association of Hashimoto's thyroiditis with thyroid hemiagenesis is quite rare and very few cases are reported in the literature. We report a case which is previously diagnosed as Graves' hyperthyroidism but developed further into Hashimoto's thyroiditis after being treated with antithyroid drugs.

## 2. Case Report

**Figure 1:**
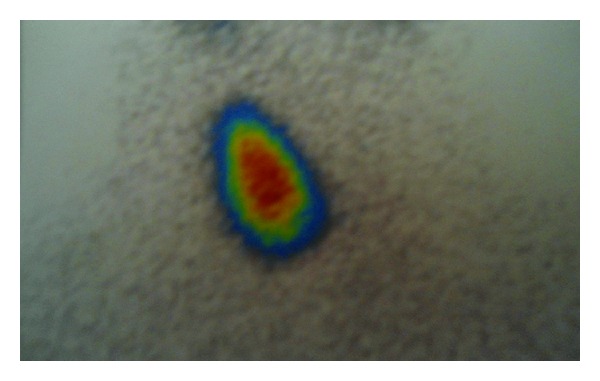
The scintiscanning documented the presence of Graves' disease within the remaining lobe.

**Figure 2:**
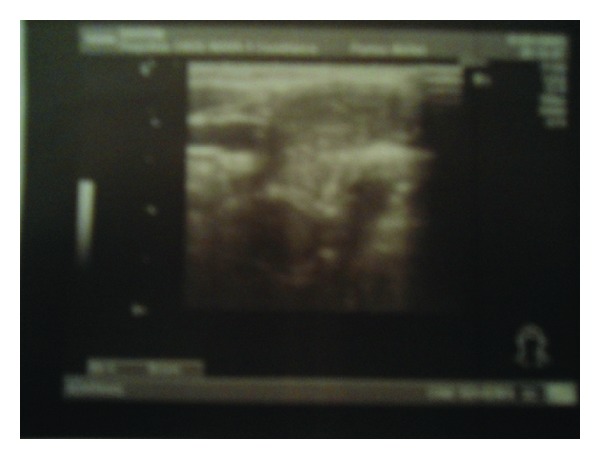
High resolution ultrasonography: right thyroid lobe having a hypoechoic, heterogenous.

**Figure 3:**
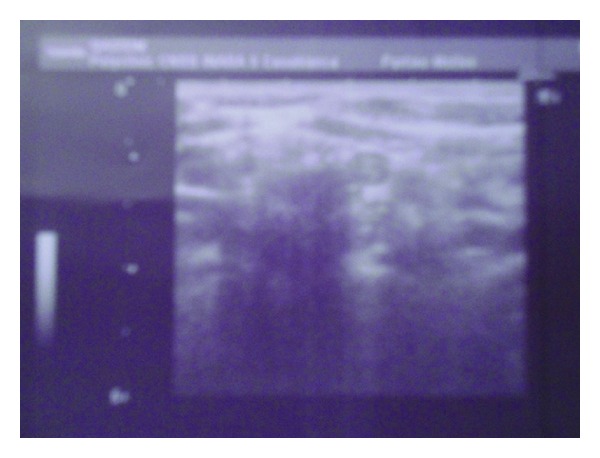
High resolution ultrasonography: isthmus and the left lobe were undetectable.

We report a 23-year-old female who was seen in December 2011 complaining of clinical hyperthyroidism manifestation, that is, weight loss, excessive sweating, palpitation, and heat intolerance. The result of thyroid function revealed that FT4 was 19.49 pg/mL (normal = 9–17 pg/mL) and TSHs was <0.022 (normal = 0.27–4.2 *μ*IU/mL). The scintiscanning documented the presence of Graves' disease within the remaining lobe (Figure 1). The antithyroid pharmacotherapy by thiamazole was prescribed by a general practitioner. However, due to symptoms of hypothyroidism, it was discontinued, two months later, and referred to our outpatient clinic. On physical examination she had a heart rate of 75/minute, blood pressure of 130/70 mmHg, and a minimally palpable right thyroid lobe. There was no proptosis, extraocular eye movements were full, and there was no eyelid retraction in both eyes. The patient has been treated with thiamazole on 3 × 10 mg/day for 2 months and the dose was discontinued after the results of FT4: 2.46 (normal = 9–17 pg/mL). The antibody test showed that antithyroglobulin (Anti-Tg), antithyroperoxidase (anti-TPO), and TSH receptor antibodies were 501.6 IU/mL (0–4.9), 594 IU/mL (0–34), and <1 IU/L (0–10), respectively. On high resolution ultrasonography right thyroid lobe was 30.6 × 25 × 52.4 mm in diameter, having a hypoechoic, heterogenous, and dystrophic parenchyma without nodules (Figure 2). Isthmus and the left lobe were undetectable (Figure 3). Based on these findings a diagnosis of thyroid hemiagenesis with Hashimoto's thyroiditis was made. The patient was being treated with Thyroxine at dose of 75 *μ*g/day. 

## 3. Discussion

The true prevalence of thyroid hemiagenesis is not known, ranges between 0.05% and 0.2% in the literature [1, 2], with an uncertain incidence; up to now 256 cases have been reported. Agenesis, for unknown reasons, concerns the left thyroid lobe in the majority of cases, 68–80% according to different reports. This is in accordance with our observation, and predominance of occurrence in women 3/1 have been most frequently reported. The isthmus is also absent in 50% of the patients [3]. Due to the development and increasing accessibility of various imaging techniques, more cases are detected. However, the etiopathogenesis of this condition, its clinical significance, impact on thyroid function, and development of associated thyroid pathologies as well as the management of patients in whom the anomaly is diagnosed are still a matter of debate [4–8]. Coexisting thyroid disorders encountered with thyroid hemiagenesis are chronic lymphocytic thyroiditis, subacute thyroiditis, nodular goiter, primary or metastatic carcinoma, and benign thyroid neoplasms with accompanying hyper-, hypo-, or euthyroidism. In a large cohort case control study of thyroid pathology in patients with thyroid hemiagenesis by Ruchala et al. [9], 40 cases of TH with various thyroid disorders have been identified: simple goiter and nonautoimmune subclinical hypothyroidism were less often observed. Patients were usually euthyroid (26 persons); however, hypothyroidism was observed in ten subjects and hyperthyroidism in the remaining four subjects. McLachlan et al. [10] identified in a review of ultrasound screening in patients with thyroid disease and, thyroid hemiagenesis, 10 cases of TH in 6242 outpatients with various thyroid disorders: 1 patient with nodular Graves' disease, 1 with Hashimoto's thyroiditis, 4 with euthyroid nodular goiters, and 2 with euthyroid multinodular goiters. Nine patients were clinically asymptomatic. TH is commonly diagnosed on thyroid scintigraphy but there are some confusing conditions. The reasons for no display of one thyroid lobe include a neoplasm, contralateral autonomous solitary thyroid nodule that is suppressing normal extranodular tissue, the inflammations and infiltrative diseases such as amyloidosis [11–14]. Therefore, it is reasonable to make a confirmation of thyroid scans with ultrasound, CT or MRI. However, ultrasonography is the best diagnostic tool as it can be performed easily everywhere, cost effectively, with no radiation exposure to the patient.

McLachlan SM regards that the existence of Hashimoto's thyroiditis is attached to immune mechanism, that is, a tolerance system which is a complex process involving central and peripheral mechanism for eliminating self-reactive lymphocytes. In addition, the role of regulatory T cells (CD4, CD25, CD28, or CD122 of T cells) which organize the self-reactive effector of T cells in the peripheral area has also been emphasized. Thyroid stimulating autoantibody is produced due to damage on self-tolerance towards TSHR. Furthermore, there is expansion of immune response toward endogenous thyroid antigen, that is, the thyroid peroxidase and thyroglobulin which consequently cause extensive lymphocytic infiltration leading to Hashimoto's thyroiditis [15]. Regulatory T cells (Treg) are a major factor in intermolecular immune response expansion, both from TSHR to TPO and Tg and alteration of hyperthyroid into Hashimoto's thyroiditis with massive thyroid lymphocytic infiltration, which causes hypothyroid state [15]. The case reported here obviously is Hashimoto's thyroiditis which points to hypothyroidism and high microsomal and thyroglobulin antibody level denoting the autoimmune process of Hashimoto's thyroiditis. It has been known that antimicrosomal antibody is found in 95% cases of Hashimoto's thyroiditis, while antithyroglobulin is found in 60% cases [16]. Diagnosis of Hashimoto's thyroiditis can be made only based on clinical manifestation of hypothyroidism along with high TSHs level as well as some tests on the occurrence of antimicrosomal antibody (AMA) and thyroglobulin antibody. Most patients with Hashimoto's thyroiditis present with diffuse thyroid enlargement and only about 10% present without thyroid enlargement, known as the atrophic form. Therefore, cytological examination of fine needle aspiration biopsy is only necessary in an uncertain case [17, 18].

## 4. Conclusion

The described case displays a very rare coincidence of hypothyroidism due to HT converted into hyperthyroidism, accompanying TH. This entity may influence the thyroid function in a different way, and hence, systematic followup and individual therapeutic management are required.
